# Koebner Phenomenon Psoriasis after Right Ankle Free Flap Reconstruction

**DOI:** 10.1055/a-2780-1435

**Published:** 2026-03-03

**Authors:** Sophie R. Persaud, Nelson A. Rodriguez-Unda

**Affiliations:** 1 Department of Plastic Surgery, Medical College of Wisconsin, Milwaukee, Wisconsin, United States

**Keywords:** Koebner phenomenon, postoperative psoriasis, free flap reconstruction, anterolateral thigh flap, case report

## Abstract

Isomorphic cutaneous lesion formation in response to trauma in patients with prior cutaneous disease is known as the Koebner phenomenon. To our knowledge, there are no reports of the phenomenon occurring after lower extremity flap reconstruction.

A 24-year-old male with no significant medical history underwent right ankle reconstruction using an anterolateral thigh flap following wide excision of a low-grade sarcoma. His recovery was mostly uneventful; however, 6 months later, he developed well-demarcated, erythematous plaques with white scales over both the flap and donor sites. The dermatology department diagnosed him with plaque psoriasis attributed to Koebnerization. Topical corticosteroids were prescribed and resulted in complete resolution within 2 days. At his 8-month follow-up, all surgical sites had healed with no evidence of recurrence.

This case highlights a rare occurrence of the Koebner phenomenon following free flap reconstruction in a patient without prior personal or familial history of psoriasis. Early detection and treatment of postoperative dermatoses in partnership with dermatology is crucial to avoiding unnecessary intervention and achieving successful outcomes without complications.

## Introduction

The Koebner phenomenon was a term first introduced for psoriatic patients in the 1870s by German dermatologist, Dr. Heinrich Koebner. 1 The phenomenon refers to the development of isomorphic lesions on the healthy skin areas of patients with preexisting cutaneous disease in response to trauma, including surgical incisions, drugs, applied pressure or friction to the skin, solar exposure, and burns. 1 2

The phenomenon is rare, and there have been no reports of the phenomenon after lower extremity free tissue transfer. Its importance relies on its differential diagnoses (avoiding costly and ineffective treatments). Here, we present the case of a young male who exhibited the Koebner phenomenon following a right ankle reconstruction after sarcoma removal. Written informed consent was obtained from the patient for the use of their clinical photographs and medical information in this publication.

## Case

**Fig. 1:**
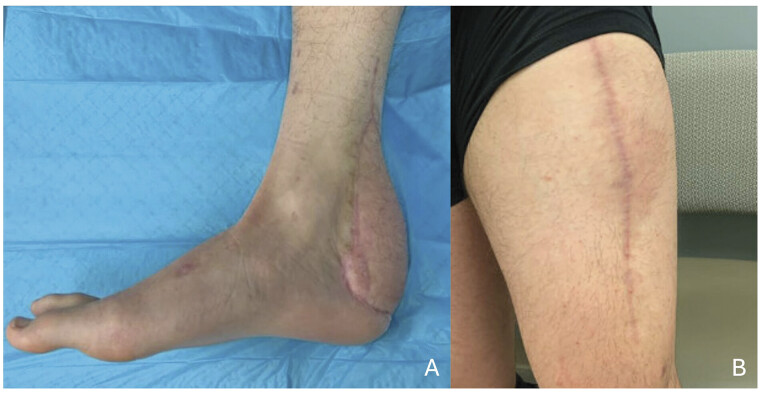
Lateral and posterior view of the right ankle prior to sarcoma removal.

A 24-year-old male patient was referred to our plastic and reconstructive surgery clinic for ankle reconstruction after the diagnosis of low-grade sarcoma over his posterior ankle Achilles tendon area ( Fig. 1 ). Besides a history of childhood eczema, he had no other medical problems. He did not use drugs or nicotine.

After multidisciplinary evaluation, the treatment consisted of staged surgical resection and negative margin confirmation by the orthopaedic surgeon with posterior left anterolateral thigh flap (ALT) reconstruction 2 to 3 weeks later.

The initial defect was measured at 6 × 6 cm over the posterior ankle with exposed talus bone and Achilles tendon. After negative margin confirmation, a contralateral left ALT measuring 8 × 9 cm was used to resurface the posterior ankle and anastomosed end-to-side to the posterior tibial artery. The veins were connected in an end-to-end fashion with a 2.5-mm venous coupler. After flap inset, in order to protect the heel from pressure, an external fixator was placed to offload any pressure over the flap.

The patient was admitted to the hospital for 7 days and discharged to continue his dangling protocol at home. He was followed up after 2-, 4-, 8-, and 12-week postoperative appointments.

**Fig. 2:**
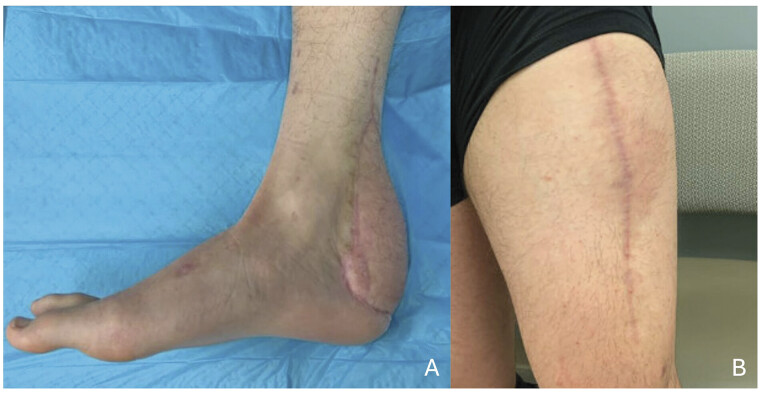
Ankle and donor site 6 months after reconstruction, showing erythema diagnosed as psoriasis, Koebner phenomenon prior to treatment.

Despite a relatively uneventful recovery, our dermatology department was consulted to see the patient 6 months after his reconstruction because he noticed a new painless rash developing over his flap and donor site thigh. Upon physical examination, both the donor and recipient sites showed signs of erythematous, well-demarcated plaques with white scales ( Fig. 2 ).

**Fig. 3:**
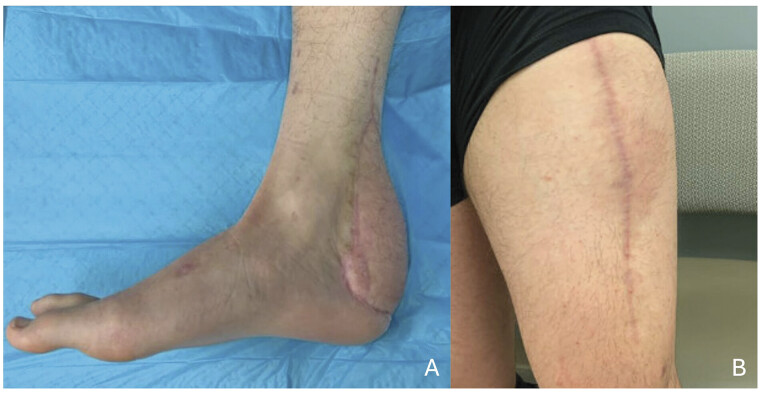
Ankle and donor site 8 months after reconstruction, showing resolution of erythema diagnosed as psoriasis, Koebner phenomenon after treatment.

The patient was given a primary diagnosis of plaque psoriasis involving 4% of his body surface area due to evidence of the Koebner phenomenon over both surgical sites. Triamcinolone acetonide 0.1% cream was prescribed, and the rash resolved 2 days later with no recurrence ( Fig. 3 ). He presented to his 8-month follow-up appointment with all incision sites healing as flat scars with minimal contact dermatitis.

## Discussion

Our case report highlights a rare occurrence of the Koebner phenomenon following free flap reconstruction in a patient without a history of psoriasis, emphasizing the importance of monitoring surgical patients for postoperative dermatoses. While the exact mechanism of Koebnerization remains unclear, multiple case reports suggest that mechanical trauma from surgery triggers the upregulation of proinflammatory cytokines and keratinocyte proliferation. 3 4 5 6 7 8

Additional differential diagnoses such as lupus, tinea corporis, and tinea incognito were also considered. Tinea corporis typically presents in young adults on the trunk with annular lesions characterized by raised margins and central clearing (“ringworm”), progressing over 1 to 3 weeks. The patient's lesions were instead well-demarcated erythematous plaques with white scale and lacked the classic ringed pattern, supporting a non-fungal etiology. Tinea incognito was ruled out based on the absence of prior topical corticosteroid or calcineurin inhibitor use, less erythematous appearance, and diffuse presentation typical of that condition. Cellulitis was excluded due to the absence of warmth, pain, or systemic symptoms such as malaise or fever; the patient had healed incisions and normal inflammatory markers. Contact dermatitis was ruled out because there was no history of new clothing, lotions, or detergents, and no vesicular changes—nor any temporal relation to topical exposures. The clinical morphology, timing after surgical trauma, and distribution at both the flap and donor sites supported a diagnosis of psoriasis with Koebner phenomenon, confirmed by dermatology. As psoriasis is primarily a clinical diagnosis, biopsy is typically reserved for cases that fail to respond to standard topical therapy, consistent with contemporary dermatologic guidelines emphasizing clinical morphology as the basis for diagnosis. 9 10

Although the rapid response to topical corticosteroids is not pathognomonic for psoriasis, the diagnosis was reinforced by the combination of classic lesion morphology (erythematous plaques with silvery scales), symmetric involvement of both surgical sites, and the absence of infection or contact irritants. Dermatology consultation corroborated the clinical impression, and the complete, sustained remission following appropriate topical therapy further supported the diagnosis.

Reports across surgical specialties have documented the Koebner phenomenon developing after reconstructive and orthopaedic procedures, even in individuals without prior dermatologic disease. In cases following breast reconstruction or flap surgery, lesions have appeared along incision lines or over donor flaps within weeks of wound healing, 4 5 11 12 13 likely triggered by localized inflammation, mechanical tension, and disruption of dermal homeostasis. Similarly, orthopaedic literature describes Koebnerization emerging after repeated fixation procedures, 11 where chronic irritation or delayed bone healing maintained an inflammatory milieu until structural stability was restored. These findings suggest that sustained microtrauma and cytokine upregulation can provoke Koebner responses in otherwise healthy skin. 14

The present case adds to this growing body of evidence by demonstrating that Koebnerization can occur simultaneously at both donor and recipient sites of a free flap, supporting the hypothesis that multisite tissue stress and wound remodeling can act as initiating stimuli. Consistent with prior reports, all lesions resolved with topical corticosteroids and without compromise to flap viability, reinforcing that timely recognition and conservative dermatologic management are sufficient for favorable outcomes.

When compared with previously reported cases of Koebner phenomenon following reconstructive procedures (e.g., breast or skin graft reconstruction), this case further illustrates that Koebnerization may arise even in patients without a personal or family history of psoriasis and may involve multiple surgical sites concurrently. Future evaluation of postoperative dermatoses should include careful exclusion of delayed cellulitis, contact dermatitis, fungal infection via KOH preparation or culture when indicated, and local tumor recurrence through imaging if warranted.

For reconstructive surgeons, Koebner phenomenon should be suspected when sharply demarcated, scaly plaques develop over well-healed incisions in the absence of erythema, drainage, or systemic symptoms. Early collaboration with dermatology enables prompt confirmation, appropriate corticosteroid therapy, and avoidance of unnecessary biopsy, antibiotics, or flap revision. Recognizing this presentation early ensures accurate diagnosis and optimizes reconstructive outcomes.
